# Characterization of the Copy Number and Variants of Deformed Wing Virus (DWV) in the Pairs of Honey Bee Pupa and Infesting *Varroa destructor* or *Tropilaelaps mercedesae*

**DOI:** 10.3389/fmicb.2017.01558

**Published:** 2017-08-22

**Authors:** Yunfei Wu, Xiaofeng Dong, Tatsuhiko Kadowaki

**Affiliations:** ^1^Department of Biological Sciences, Xi’an Jiaotong-Liverpool University Suzhou, China; ^2^School of Life Sciences, Jiangsu Normal University Xuzhou, China

**Keywords:** honey bee, ectoparasitic mite, deformed wing virus, host–pathogen interaction, viral replication

## Abstract

Recent honey bee colony losses, particularly during the winter, have been shown to be associated with the presence of both ectoparasitic mites and Deformed Wing Virus (DWV). Whilst the role of *Varroa destructor* mites as a viral vector is well established, the role of *Tropilaelaps mercedesae* mites in viral transmission has not been fully investigated. In this study, we tested the effects that *V. destructor* and *T. mercedesae* infestation have on fluctuation of the DWV copy number and alteration of the virus variants in honey bees by characterizing individual pupae and their infesting mites. We observed that both mite species were associated with increased viral copy number in honey bee pupae. We found a positive correlation between DWV copy number in pupae and copy number in infesting mites, and the same DWV type A variant was present in either low or high copy number in both honey bee pupae and infesting *V. destructor*. These data also suggest that variant diversity is similar between honey bee pupae and the mites that infest them. These results support a previously proposed hypothesis that DWV suppresses the honey bee immune system when virus copy number reaches a specific threshold, promoting greater replication.

## Introduction

Large-scale honey bee colony loss is increasingly being reported across North America and Europe (Goulson et al., 2015). Pollination by honey bees is critical to a functional ecosystem and also production for many crops (Klein et al., 2007; Aizen and Harder, 2009). Prevention of honey bee losses is therefore vitally important to both the apiculture and agriculture industries. Although many potential causes for the decline have been proposed, pathogens and parasites of honey bees, particularly ectoparasitic mites are considered major threats to colony health (Evans and Schwarz, 2011; Goulson et al., 2015). One such mite species, *Varroa destructor*, is globally distributed (except Australia) and causes both abnormal brood development and brood death in honey bee colonies (Rosenkranz et al., 2010). These effects are elicited through mites feeding on hemolymph and also by spreading honey bee viruses, particularly Deformed Wing Virus (DWV) (de Miranda and Genersch, 2010; Martin et al., 2012).

In many Asian countries, *Apis mellifera* colonies are also infested with another ectoparasitic mite, *Tropilaelaps mercedesae*, and this mite species often co-exist with *V. destructor* in a single colony (Anderson and Morgan, 2007; Luo et al., 2011). *T. mercedesae* has a shorter life cycle than *V. destructor*, without a phoretic phase involving honey bee adults. It also produces a higher number of offspring compared to *V. destructor* (Anderson and Roberts, 2013). However, these two emerging parasites of *A. mellifera* share many characteristics (Anderson and Roberts, 2013). For example, their reproductive strategies are similar (Sammataro et al., 2000) and both are vectors for DWV (Yue and Genersch, 2005; Dainat et al., 2009; Forsgren et al., 2009). Finally, *T. mercedesae* and *V. destructor* infestations have similar negative impacts on *A. mellifera* colonies (Dainat et al., 2012; Khongphinitbunjong et al., 2016; Nazzi and Le Conte, 2016). Although *T. mercedesae* is currently limited to Asia, the species has the potential to spread and establish in Europe and North America via the global trade in honey bees.

Deformed Wing Virus is phylogenetically classified to the *Iflaviridae* family in the *Picornavirales* order (Lanzi et al., 2006). Like other RNA viruses, DWV exists as a quasi-species with three major variants identified (type A, B, and C) (Mordecai et al., 2016a,b). The first characterized DWV and Kakugo virus have now been classified as a type A, while Varroa Destructor Virus-1 (VDV-1) is a type B (Mordecai et al., 2016a). DWV type C has recently been described and is phylogenetically distinct from both A and B types (Mordecai et al., 2016b). Recently, DWV has been successfully reproduced by injecting the *in vitro* synthesized viral genome RNA to honey bee pupae (Lamp et al., 2016) and the structure of DWV has also been revealed by cryo-EM and X-ray crystallography (Organtini et al., 2017; Škubník et al., 2017).

Previous studies have reported that honey bee colony losses frequently associate with *V. destructor* infestation, as well as increased DWV prevalence and copy number (Highfield et al., 2009; Dainat et al., 2012; Nazzi and Le Conte, 2016). This leads to malformed wings and reduced lifespans during mite infestations (de Miranda and Genersch, 2010; Rosenkranz et al., 2010). The underlying molecular mechanisms for these pathologies as well as the high copy number infection are not yet understood but several different hypotheses have been proposed. These include (1) *V. destructor* induces immune-suppression in the honey bee, stimulating DWV replication (Yang and Cox-Foster, 2005), (2) *V. destructor* selectively amplifies more virulent DWV strains (Martin et al., 2012), (3) DWV suppresses the honey bee immune system when virus copy number reaches a specific threshold, promoting greater replication (Nazzi et al., 2012; Di Prisco et al., 2016), and (4) Direct injection of the mixed variants of DWV to hemolymph of honey bee results in replication of the selective variant to high copy number (Ryabov et al., 2014). However, immune-suppression of honey bee by *V. destructor* infestation has not been observed in other studies (Gregorc et al., 2012; Kuster et al., 2014).

In this study, we compared the effects of *V. destructor* and *T. mercedesae* infestation on DWV prevalence and copy number in honey bee pupae by individually analyzing pupae and infesting mites from the same capped brood cell. Together with sequencing of DWV in paired pupae and mites, we use these data to discuss about the potential mechanisms for the stimulation of DWV replication by *V. destructor* and *T. mercedesae* in honey bees. Many previous studies have analyzed the pooled samples of honey bees and mites, and the results are useful to understand the general degree of DWV infection and identify the dominant variant in the colony. However, our analyses were aimed to understand micro dynamics of DWV population (as well as flow of DWV) between the pairs of honey bee pupa and infesting mite.

## Materials and Methods

### Sample Collection

A single *A. mellifera* colony, sourced from a local beekeeper, was brought to Xi’an Jiaotong Liverpool University in April 2016. Honey bee pupae with purple eyes were collected by opening capped brood cells. Most pupae were free from mites in the period April 20-23 2016, with only a small number infested with *V. destructor*. During May 20-23 2016, we collected further pupae and found that 33% of the capped brood cells were now infested with *V. destructor* and none of them contained *T. mercedesae*. When purple eyed-pupae were infested by *V. destructor*, both were sampled simultaneously and stored separately. A second *A. mellifera* colony infested with *T. mercedesae* was brought in October 2016. Purple eyed-pupae, with or without infesting *T. mercedesae*, were collected from November 1–8 2016, as described above (50% of capped brood cells contained *T. mercedesae). V. destructor* was absent in all of the opened capped brood cells.

### RT-PCR Analysis of DWV

Total RNA was isolated from individual pupae and mites using Total RNA Extraction Reagent (GeneSolution), according to the manufacturer’s instructions. Glycogen (1 μg) was added to facilitate isopropanol precipitation of the mite RNA samples. Reverse transcription (RT) reactions were carried out using 1 μL of total RNA, random primers (TOYOBO), ReverTra Ace (TOYOBO), and RNase Inhibitor (Beyotime). RNase H (Beyotime) was added to digest RNA in RNA/cDNA heteroduplex after cDNA synthesis. These RT products were used for subsequent RT-PCR and qRT-PCR. RT-PCR was conducted to assess DWV infection in sampled pupae and mites using KOD-FX (TOYOBO) and the DWV primer set #1 (Supplementary Table S1) using the conditions 2 min at 94°C, followed by 32 cycles of 10 s at 98°C, 20 s at 55°C, and 30 s at 68°C, with a final 3 min extension at 68°C. PCR products were analyzed on a 2% agarose gel. PCR targeting *A. mellifera* and *T. mercedesae EF-1α*, and *V. destructor β-actin* mRNAs (Supplementary Table S1), were used as controls to verify successful RT (Supplementary Figure S1).

### qRT-PCR Analysis of DWV Copy Number

We performed qRT-PCR using a Hieff^TM^^®^ qPCR SYBR Green Master Mix (Low Rox Plus, Yesen) and DWV primer set #2 (Supplementary Table S1) with a QuantStudio5 Real-Time PCR System (Thermo Fischer). To perform absolute quantification of DWV RNA, we first prepared standard curves that corresponded to DWV target RNA. Target DNA was prepared by PCR, followed by purification with a AxyPrep^TM^ PCR cleanup kit (Axygen). DNA concentration was measured using a Nanodrop 2000 spectrophotometer (Thermo Fischer) to calculate copy number using the formula Copy number = DNA concentration (ng/μL) × 6.02 × 10^23^ (copies/mol)/length (bp) × 6.6 × 10^11^ (ng/mol), in which 6.6 × 10^11^ ng/mol is the average molecular mass of one base pair and 6.022 × 10^23^ copies/mol is Avogadro’s number. Linear standard curves were then generated using target DNA of 10^1^–10^9^ copy number per reaction, followed by plotting of Ct values against log copy number values. After RT, the copy number of target RNA in a sample was estimated using the standard curve. The amount of cDNA added to each reaction was normalized by the Ct values of either *A. mellifera, V. destructor*, or *T. mercedesae 18S rRNA*.

### Sequencing of RT-PCR Products

PCR products obtained by RT-PCR were purified and directly sequenced by Sanger sequencing. To sequence the 4 kb DWV RNA region encoding partial sequence for Lp, helicase, and all of the VP proteins, we performed PCR using DWV primer set #3 (Supplementary Table S1) using the conditions 2 min at 94 °C, followed by 35 cycles of 10 s at 98°C, 20 s at 55°C, and 3.5 min at 68°C, with a final 5 min extension at 68°C. These PCR products were sequenced as previously described, and categorized into DWV viral subtypes (A, B, and C).

### Construction of Bayesian Phylogenetic Trees

Prior to phylogenetic analysis, we aligned nucleotide sequences using Mafft (v7.307) (Katoh and Standley, 2013) and then used Gblocks (v0.91b) (Castresana, 2000) to automatically eliminate divergent regions or gaps. The best fit nucleotide substitution models were determined for the alignments by Jmodeltest (v2.1.10) with parameters set to “-g 4 -i -f -AIC -BIC -a” (Darriba et al., 2012). Mrbayes (v3.2.3) (Ronquist and Huelsenbeck, 2003) was run with the parameters set to 1,000,000 (1 sample/100 generations) until split frequencies were below 0.01. The first 25% of samples for each run were designated as the burn-in. DWV type A strain (NC_004830.2), DWV type B strain (NC_006494.1), DWV type C strain (CEND01000001.1), and Sacbrood virus (NC_002066.1) were used as references.

## Results

### Effects of Infestation by Two Mite Species on DWV Infection in Honey Bee Pupae

**FIGURE 1:**
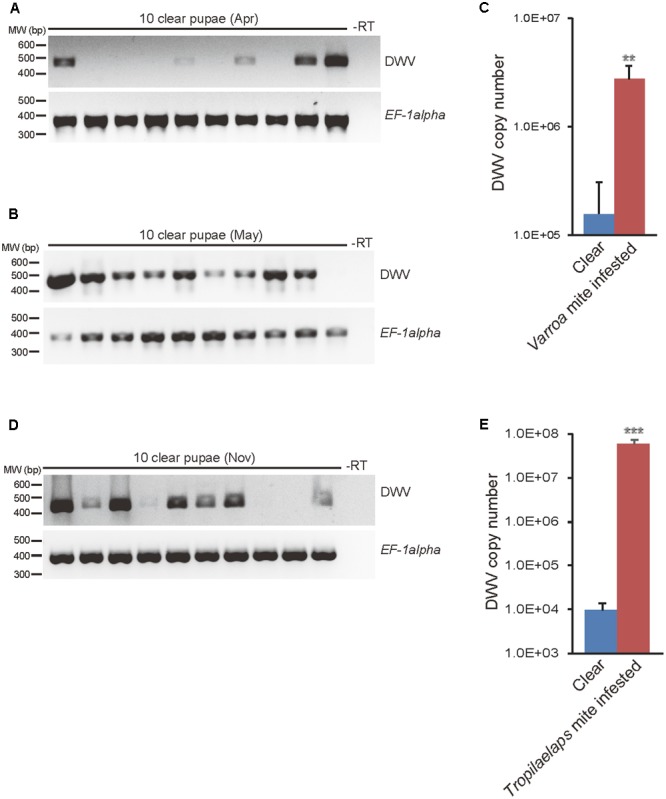
Detection and quantification of DWV in mite-infested and clear honey bee pupae. DWV was detected using RT-PCR in 10 purple eyed-pupae without direct *V. destructor* infestation sampled in **(A)** April 2016 when the mite infestation was low and in **(B)** May 2016 when the infestation was high. Honey bee *EF-1alpha* mRNA was used as an endogenous positive control and water (-RT) used as a negative control. The position of a 400–600 bp DNA molecular weight marker (MW) is shown (left). **(C)** DWV copy number in purple eyed-pupae from *V. destructor* infested (*n* = 23) or clear (*n* = 23) capped cells was measured by qRT-PCR. The mean value with error bar (±SEM) is indicated for each sample. The copy number is higher in the mite infested pupae (^∗∗^) (*P* < 0.0065, two-tailed Welch’s *t*-test). **(D)** DWV was detected by RT-PCR in 10 purple eyed-pupae without direct *T. mercedesae* infestation sampled in November 2016 when the mite infestation was high. **(E)** DWV copy number in purple eyed-pupae from *T. mercedesae* infested (*n* = 28) or clear (*n* = 19) capped cells was measured by qRT-PCR. The mean value with error bar (±SEM) is indicated for each sample. The copy number is higher in the mite infested pupae (^∗∗∗^) (*P* < 0.000013, two-tailed Welch’s *t*-test).

In April 2016, we collected purple eyed-honey bee pupae (6–8 days after capping) from a single colony. In this initial screen, there were almost no mites but approximately 61% (11 out of 18) of the honey bee pupae were infected with DWV when analyzed using RT-PCR (**Figure 1A** for analysis of 10 pupae).

In a second collection from the same colony in May 2016, we found that *V. destructor* was more abundant and 94% (17 out of 18) of pupae without infesting mites in the capped brood cells were DWV positive (**Figure 1B** for analysis of 10 pupae) (*P* < 0.041; two-tailed Fisher’s exact test). We also examined pupae from capped brood cells that were infested with *V. destructor* and found that 100% were positive for DWV infection (30 out of 30). We compared the copy number of DWV from pupae in mite infested cells to pupae without direct mite infestation. As shown in **Figure 1C**, mite infested pupae had a higher DWV copy number compared to those without infestation.

In November 2016, we also collected purple eyed-honey bee pupae from a colony infested with *T. mercedesae*. In this colony, 65% (13 out of 20) of pupae in capped brood cells without infesting mites were positive for DWV (**Figure 1D** for analyzing 10 pupae). We again collected pupae in capped brood cells infested by *T. mercedesae* (100% positive for DWV, 31 pupae) and compared their DWV copy number to pupae without direct mite infestation. As shown in **Figure 1E**, mite infested pupae had a DWV higher copy number than those without infestation.

### DWV Copy Number between Honey Bee Pupae and Infesting Mites

**FIGURE 2:**
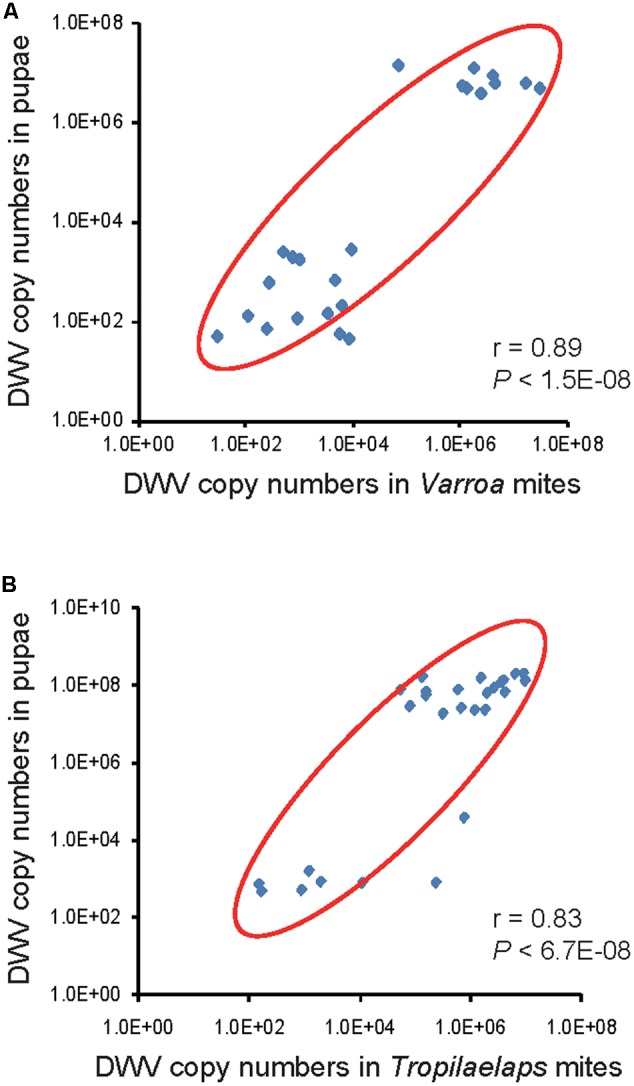
Correlation of DWV copy number in honey bee pupae and their infesting mites. **(A)** DWV copy number in individual honey bee pupae and infesting *V. destructor* or **(B)** *T. mercedesae* are plotted on the *Y*- and *X*-axis, respectively. Red ovals represent coverage of more than 87% of the data. The Pearson correlation values and *P*-values are also shown.

We next measured and compared DWV copy number in 23 honey bee pupa/infesting *Varroa* mite pairs isolated from the same capped brood cells. We found a linear correlation between DWV copy number in the pupa and infesting mite. Specifically, we observed that the samples grouped into two clusters that were either low or high DWV copy number (**Figure 2A**). There were more pairs of pupa/mite in the cluster with low copy number of DWV. We also performed the same analysis with 28 honey bee pupa/*T. mercedesae* capped brood cell pairs; a linear correlation and the presence of two copy number clusters was also found in this mite species (**Figure 2B**). In contrast to *V. destructor* above, there were more pairs of pupa/mite in the cluster with high copy number of DWV in case of *T. mercedesae* infestation.

### DWV Variants and Types Found in Honey Bee Pupae and Mites

In order to determine the strains of DWV isolates in our study, we directly sequenced RT-PCR products containing the partial sequences for VP2 and VP1, and full sequence of VP4, from 24 honey bee pupa/*V. destructor* pairs isolated from matched capped brood cells.

**FIGURE 3:**
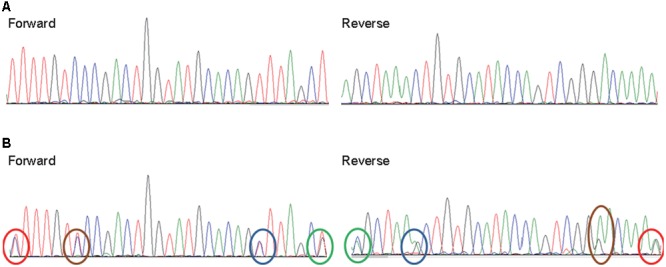
Infections of single and multiple DWV variants in honey bee pupae and infesting mites. Representative Sanger sequencing electrograms (both forward and reverse) of RT-PCR products reveal both single variant and multiple DWV variants infections in honey bee pupae and infesting *V. destructor* or *T. mercedesae*. **(A)** Only single peaks are present at all positions of this sample but **(B)** two peaks are present at four positions in the second sample (marked by red, brown, blue, and green ovals in both forward and reverse sequences).

Examination of the Sanger sequencing electrograms demonstrated that both pupae and mites could be infected by either a single (**Figure 3A**) or multiple DWV variants (**Figure 3B**). For example, in the sequence shown in **Figure 3A**, single peaks are present at all positions in both forward and reverse sequencing reactions. However, in other samples, multiple peaks are present at equivalent positions in both forward and reverse sequencing reactions (**Figure 3B**), demonstrating that these multiple peaks are not sequencing artifacts. Nevertheless, it is still possible that deep transcriptome sequencing may reveal the presence of very low prevalence variant(s) in infected pupae or mites that appear infected with a single variant. In our analysis, we found that eight pairs of pupa and *Varroa* mite shared infection by a single DWV variant, although the variant observed differed between pairs. There were three pairs in which pupae were infected by a single variant and the mites infected by multiple variants. Conversely, there were three pairs in which pupae were infected by multiple variants and the mites a single variant. Finally, the remaining 10 pupa and mite pairs were infected by multiple variants in each (**Table 1**).

**Table 1:** Profile of DWV infection in the honey bee pupae and the infesting *Varroa* mites.

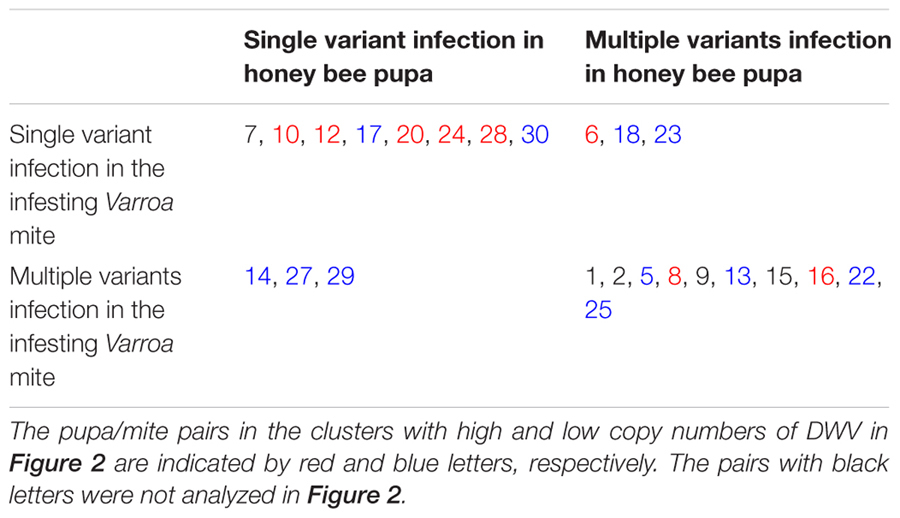

We also analyzed DWV sequences in 17 honey bee pupa/*T. mercedesae* pairs. We found that two pairs of pupa and mite shared infection by single DWV variants, although the viral variant again varied between the pairs. There was a single pupa and mite pair in which the pupa was infected by a single variant and the mites infected with multiple variants. There was also one pair in which the pupa was infected by multiple variants and the mites infected by a single variant. The remaining 13 pairs were infected by multiple variants in both pupae and mites (**Table 2**).

**Table 2:** Profile of DWV infection in the honey bee pupae and the infesting *Tropilaelaps* mites.

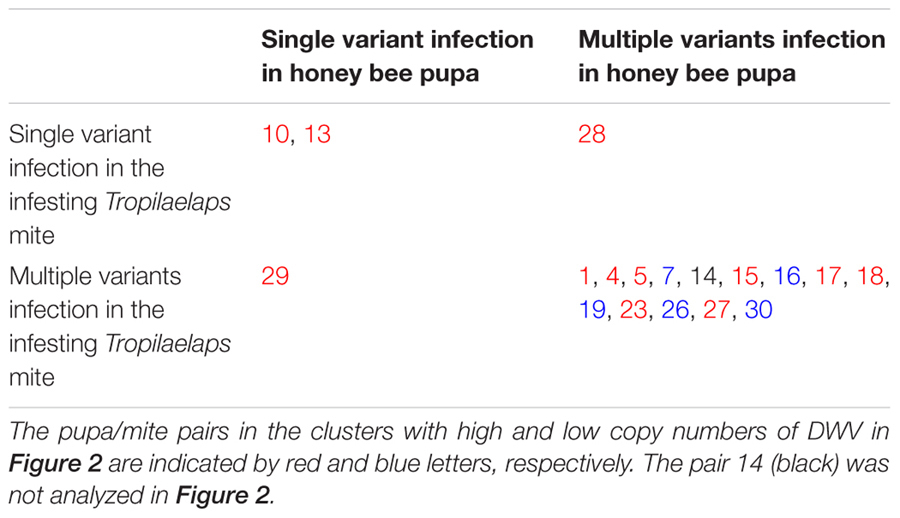

**FIGURE 4:**
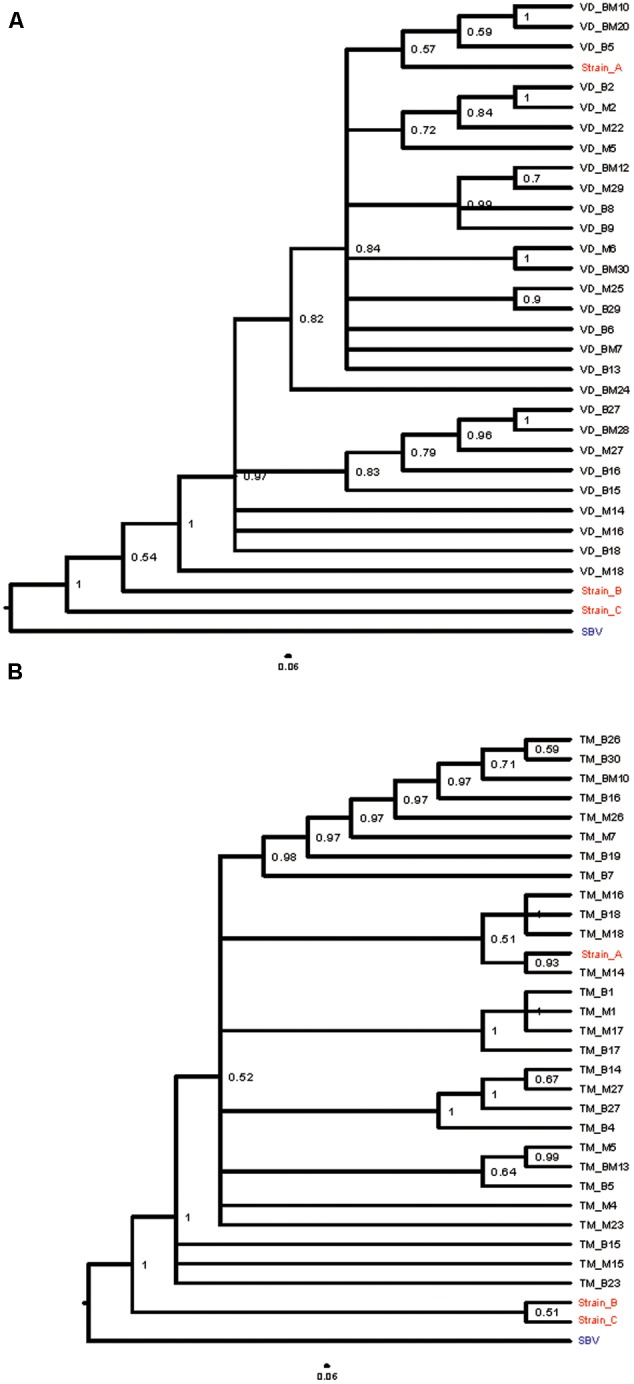
Bayesian phylogenetic trees of DWV isolates based on a 434 nt region encoding viral structural proteins. **(A)** Bayesian phylogenetic trees of DWV isolates from *V. destructor* infested honey bee pupae (VD_B) and matched mites (VD_M), and **(B)** *T. mercedesae* infested honey bee pupae (TM_B) and mites (TM_M), were constructed based on partial VP2 and VP1, and full VP4 sequences. Cases in which a pupa and infesting mites were infected by an identical single DWV variant are indicated as either VD_BM or TM_BM. Posterior probabilities are shown at the corresponding node of each branch. DWV type A, B, and C strains (with red letter) were included for analysis as references and SBV (Sac brood virus) with blue letter was used as an outgroup.

**FIGURE 5:**
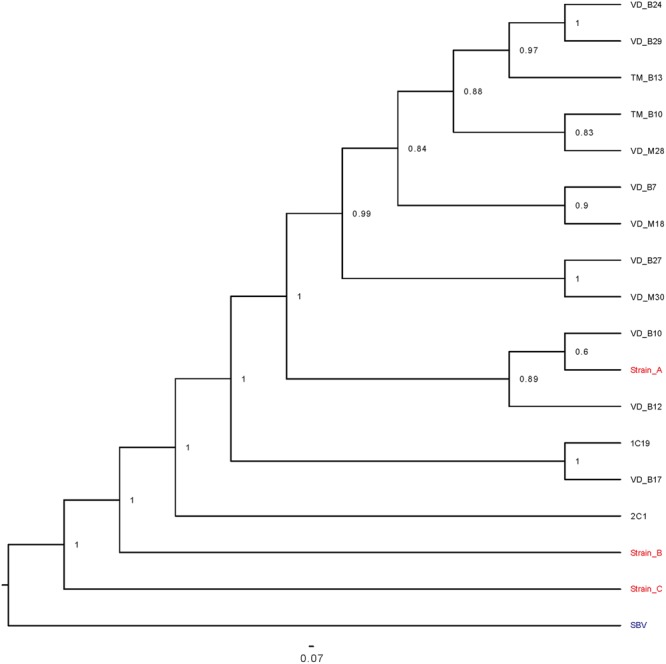
Bayesian phylogenetic tree of representative DWV isolates based on a 3633 nt region encoding viral structural proteins and parts of the Lp and helicase proteins. A Bayesian phylogenetic tree of representative DWV isolates was constructed based on partial Lp and helicase sequences, and full VP sequences. 1C19 and 2C1 were isolated from the mite-free honey bee pupae.

Phylogenetic relationships estimated using these sequence (Accession number: MF134306-MF134370), and published DWV type A, B, and C sequences, indicated that all the DWV isolates in our study were more related to DWV type A rather than types B or C (**Figures 4A,B**). Because most posterior probabilities in the trees were less than 1, we additionally sequenced a 4 kb region of the viral genome containing part of the Lp sequence, all of the VP genes, and part of the helicase gene, from 14 representative samples (*V. destructor, T. mercedesae*, mite-infested pupae, and infestation-free pupae) in total. A phylogenetic tree was then estimated from these data (Accession number: MF134371-MF134383). As shown in **Figure 5**, all isolates are distinct from DWV types B and C and cluster together with type A. Although 2C1 outgroups the other isolates, including the type A (**Figure 5**), the viral genome sequence encoding the entire polyprotein, as well as parts of the 5′ and 3′ UTRs (Accession number: MF036686) shows higher identity to type A (96%) rather than types B (84%) or C (80%).

### Association between DWV Variants and Copy Number in Honey Bee Pupae and Infesting Mites

As shown in **Table 1**, 19 honey bee pupa and infesting *V. destructor* pairs were previously classified into high and low DWV copy number clusters (**Figure 2**). Among these, the same DWV variant was present in both the #10 pupa/mite pair and #14 pupa (**Figure 4**). However, the #10 and #14 pairs were classified into high and low copy number clusters, respectively (**Table 1**). A similar pattern was shown in the #6 mite sample (high copy number) and #23 mite sample (low copy number). These results demonstrate that the same DWV type A variant can result in either low or high copy number in both honey bee pupae and infesting *V. destructor*. As all low copy number pairs of pupa and infesting *T. mercedesae* were infected by multiple DWV variants (**Table 2**), a similar characterization was not possible for this species.

## Discussion

### *V. destructor* and *T. mercedesae* Infestation Increases DWV Copy Number in Honey Bee Pupae

Our results demonstrate that an increase in the *V. destructor* population of a colony enhances the prevalence of DWV infection in honey bee pupae, even in pupae without direct mite infestation. Furthermore, direct infestation of pupae with either *V. destructor* or *T. mercedesae* dramatically increased DWV copy number of the pupa. These results support previous reports that *V. destructor* and *T. mercedesae* function as vectors for DWV and promote transmission in honey bees (Shen et al., 2005; Yue and Genersch, 2005; Forsgren et al., 2009). Our study indicates that both mite species are equally effective at increasing DWV copy number in infested honey bee pupae.

These data raise the question as to the source of DWV present in honey bee pupae without direct mite infestation. DWV could be present in larval food (Yue and Genersch, 2005; Singh et al., 2010) and that copy number may increase if honey bee workers have high DWV copy number due to mite infestation. Furthermore, *V. destructor* and *T. mercedesae* carrying DWV may enter brood cells, feed on hemolymph, but then leave before the cell is capped. Vertical transmission of DWV from the queen may also occur and the mite infestation of drone would enhance this process by producing the semen containing high titer of DWV (Chen et al., 2006; Yue et al., 2007; de Miranda and Fries, 2008).

The same question as to the initial source of DWV also applies to virus found in *V. destructor* and *T. mercedesae.* DWV may again be vertically transmitted from the mother mites and this hypothesis is supported by our recent study demonstrating that *T. mercedesae* males and nymphs have high DWV copy numbers that are the same as females (Dong et al., 2017). In addition, DWV could be horizontally transmitted from infested pupae (for both *V. destructor* and *T. mercedesae*) or adult workers (for *V. destructor* only).

We have confirmed that there is a linear correlation between DWV copy number in honey bee pupae and the mite individual that infests them, supporting previous studies (Forsgren et al., 2009; Kielmanowicz et al., 2015). For both *V. destructor* and *T. mercedesae*, pupa/mite pairs clustered into two groups containing either low or high DWV copy number. These results suggest that DWV replication in honey bee pupae is positively regulated relative to the amount of DWV introduced by infesting mites. As different DWV variants were found in the honey bee pupa/mite pairs (**Tables 1, 2**), it seems that DWV does not simply exchange/flow between pupa and mite. We found that both honey bee pupae and mites can be infected by either a single variant or multiple DWV variants before the mite enters the brood cell containing the fifth instar larva. In fact, we found that 19 out of 20 pupae were infected by multiple DWV variants, despite no direct *V. destructor* infestation.

### *V. destructor* and *T. mercedesae* Mediated Control of DWV Replication in Honey Bee Pupae

The fact that *V. destructor* infestation increases DWV copy number in honey bees was well established (Shen et al., 2005; Yue and Genersch, 2005) and four major mechanisms have been previously proposed, (1) *V. destructor* induces immune-suppression in honey bees, stimulating DWV replication (Yang and Cox-Foster, 2005), (2) Replication-active more virulent DWV strains are selected for by *V. destructor* infestation (Martin et al., 2012), (3) DWV induces immune-suppression in honey bees at a threshold copy number (Nazzi et al., 2012; Di Prisco et al., 2016), and (4) Direct injection of the mixed variants of DWV to hemolymph of honey bee results in replication of the selective variant to high copy number (Ryabov et al., 2014). Our results seem to be inconsistent with the first and second proposed mechanisms.

If mite-mediated immune-suppression in honey bees occurs irrespective of DWV copy number in infesting mites, the copy number found in the paired honey bee pupae would not correlate with that in the mites. However, we found a strong correlation in copy number between mites and pupae, suggesting a relationship. Furthermore, even though the DWV isolates in our study were classified as the virulent type A (Mordecai et al., 2016a,b), the same variants were present in low and high copy number clusters in both honey bee pupae and mites. The patterns of DWV infection with either single or multiple variants in honey bee pupae and infesting mites appear to be the same (**Tables 1, 2**), suggesting that *V. destructor* and *T. mercedesae* may not select for a specific type A variant. This observation is similar to previous report suggesting that a single type of virulent DWV is amplified in honey bee pupae, despite the infesting *V. destructor* contains a diverse population of DWV strains (Ryabov et al., 2014). However, this may not be the case if other types (B and C) co-exist (Mordecai et al., 2016a). Based on several reports, both types A and B can be equally virulent (McMahon et al., 2016; Mordecai et al., 2016a). Selection of specific virulent DWV types/variants may depend on the extent of replication in the mites, although the replication mechanism of DWV in honey bees and mites is not yet fully understood.

Our data are most consistent with the third proposed mechanism: that there is immune-suppression at a threshold copy number. This support derives from our observation that DWV replication in honey bee pupae appears to depend on the copy number of DWV introduced by infesting mites. Honey bee pupae may be able to suppress DWV replication if only low levels of virus are introduced by the mite, keeping DWV copy number in the pupae relatively low. Having two major clusters of the pupa/mite pairs with low and high (but not medium) copy number of DWV (**Figure 2**) also supports this hypothesis. Surprisingly, the same results were basically obtained with two different mite species, suggesting that the common mechanism is likely to operate. If this model is correct, the dominant DWV variant present in the mite should also be the dominate type in the pupa of each high DWV copy number pupa/mite pair. Our study suggests that this is the case for both *V. destructor* and *T. mercedesae.* For example, the same DWV variant was present in all pupa/*V. destructor* pairs if they were singly infected, irrespective of copy number. In pair #6, the single variant present in the mite dominated the multiple variants found in the honey bee pupa. Both pupae and *V. destructor* in the pairs of #8 and #16 were infected by multiple DWV variants, although the dominant variant was shared between them (**Table 1**). The same pattern also occurs in pairs of pupa/*T. mercedesae* (**Table 2**). These findings support the hypothesis that DWV replication in honey bee pupae is positively regulated relative to the amount of DWV introduced by infesting mites. This may be due to immune-suppression in the pupae induced by high DWV copy number (Nazzi et al., 2012; Di Prisco et al., 2016). Our results do not fit well to the fourth proposed hypothesis above. Many individuals of *V. destructor* and *T. mercedesae* were infected by multiple variants and most of the mite-infested honey bee pupae were infected by multiple variants at either low or high copy number (**Tables 1, 2**). This hypothesis may also depend on the copy number of DWV injected as the third proposed mechanism. Our discussion above is based on the results obtained with 23 pupa/*V. destructor* and 29 pupa/*T. mercedesae* pairs, and thus analyzing more pairs in multiple colonies will be necessary to strengthen our arguments.

As the copy number of DWV in *V. destructor* or *T. mercedesae* is the critical factor determining the extent of DWV replication in infested honey bees, it is important to understand the transmission and replication mechanisms of DWV in the mites as these may differ from those in honey bee. The DWV population is extremely heterogeneous, with many variants and recombinants between different types in both honey bees and mites, even in the single colony. This viral heterogeneity may emerge because there are multiple hosts for the virus (honey bee, *V. destructor*, and *T. mercedesae*). It is thus important to understand how actively the different variants and recombinants replicate in honey bee and mite.

## Author Contributions

YW and XD conducted all experiments. YW and TK designed the experiments and wrote the manuscript.

## Conflict of Interest Statement

The authors declare that the research was conducted in the absence of any commercial or financial relationships that could be construed as a potential conflict of interest.

## Supplementary Material

FIGURE S1 Original images of agarose gels shown in **(A–C)**. Honey bee EF-1alpha mRNA and DWV were detected by RT-PCR. Water (-RT) was used as the negative control.
