# Supplementary figures and images for: Characterization of the Copy Number and Variants of Deformed Wing Virus (DWV) in the Pairs of Honey Bee Pupa and Infesting Varroa destructor or Tropilaelaps mercedesae
Source: Front Microbiol. 2017 Aug 22;8:1558. doi: 10.3389/fmicb.2017.01558 (PMC5572262; doi:10.3389/fmicb.2017.01558)

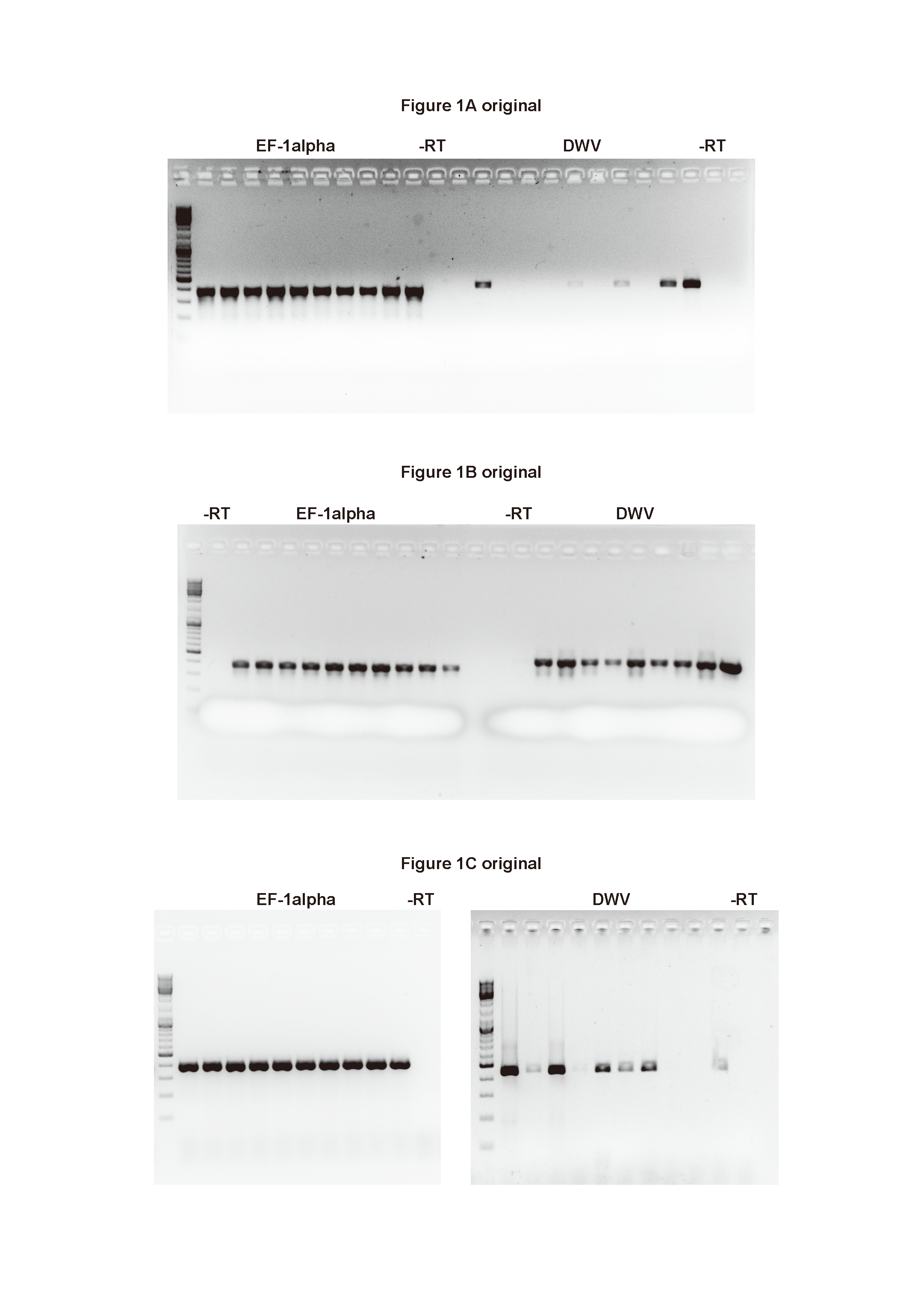

Supplement: FIGURE S1 — Original images of agarose gels shown in (A–C). Honey bee EF-1alpha mRNA and DWV were detected by RT-PCR. Water (-RT) was used as the negative control. [file Image_1.TIF]
