# Supplementary material for: Characterization of the Copy Number and Variants of Deformed Wing Virus (DWV) in the Pairs of Honey Bee Pupa and Infesting Varroa destructor or Tropilaelaps mercedesae
Source: Front Microbiol. 2017 Aug 22;8:1558. doi: 10.3389/fmicb.2017.01558 (PMC5572262; doi:10.3389/fmicb.2017.01558)
Supplement: Supplementary file 2 [file Table_1.DOCX]

Supplementary Table 1 Primer sets used for RT-PCR and qRT-qPCR

| Target | Forward (F) and reverse (R) primer sequences (5’-3’) | Expected product size (bp) | References |
| --- | --- | --- | --- |
| RT-PCR | | | |
| DWV #1 | (F) ATTGTGCCAGATTGGACTAC | 435 | Benjeddou et al., 2001 |
|  | (R) AGATGCAATGGAGGATACAG |  |  |
| DWV #3 | (F) GATCGCTGAACGTTGTACGC | 3633 | This study |
|  | (R) ATACCCAAGCACTTGCCTCC |  |  |
| *A. mellifera EF-1α* | (F) TGCAAGAGGCTGTTCCTGGTGA | 378 | Kojima et al., 2011 |
|  | (R) CGAAACGCCCCAAAGGCGGA |  |  |
| *V. destructor β-actin* | (F) TCGTACGAGCTTCCCGACGGT | 331 | Yang et al., 2013 |
|  | (R) GGGAGGCAAGGATGGAACCGC |  |  |
| *T. mercedesae EF-1α* | (F) ATTCCGGTAAGTCAACCACCAC | 150 | Dong et al., 2017 |
|  | (R) GCTCGGCCTTCAGTTTGTCCAA |  |  |
| qRT-qPCR | | | |
| DWV #2 | (F) TTCATTAAAGCCACCTGGAACATC | 136 | Locke et al., 2012 |
|  | (R) TTTCCTCATTAACTGTGTCGTTGA |  |  |
| *A. mellifera 18S rRNA* | (F) ACCACATCCAAGGAAGGCAG | 112 | This study |
|  | (R) ACTCATTCCGATTACGGGGC |  |  |
| *V. destructor 18S rRNA* | (F) GTGAAACCGCGAATGGCTCA | 147 | This study |
|  | (R) TCCGAAGACATGGTTTGCACT |  |  |
| *T. mercedesae 18S rRNA* | (F) CCTTCGGACTTACGGTGACG | 188 | This study |
|  | (R) TATGTGGTCGCCGTTTCTCA |  |  |

**Benjeddou, M., Leat, N., Allsopp, M. and Davison, S.** (2001). Detection of acute bee paralysis virus and black queen cell virus from honeybees by reverse transcriptase pcr. *Appl Environ Microbiol* **67**, 2384-2387.

**Dong, X., Armstrong, S. D., Xia, D., Makepeace, B. L., Darby, A. C. and Kadowaki, T.** (2017). Draft genome of the honey bee ectoparasitic mite, Tropilaelaps mercedesae, is shaped by the parasitic life history. *Gigascience* **6**, 1-17.

**Kojima, Y., Toki, T., Morimoto, T., Yoshiyama, M., Kimura, K. and Kadowaki, T.** (2011). Infestation of Japanese native honey bees by tracheal mite and virus from non-native European honey bees in Japan. *Microb Ecol* **62**, 895-906.

**Locke, B., Forsgren, E., Fries, I. and de Miranda, J. R.** (2012). Acaricide treatment affects viral dynamics in Varroa destructor-infested honey bee colonies via both host physiology and mite control. *Appl Environ Microbiol* **78**, 227-235.

**Yang, B., Peng, G., Li, T. and Kadowaki, T.** (2013). Molecular and phylogenetic characterization of honey bee viruses, Nosema microsporidia, protozoan parasites, and parasitic mites in China. *Ecol Evol* **3**, 298-311.
